# Polytomy identification in microbial phylogenetic reconstruction

**DOI:** 10.1186/1752-0509-5-S3-S2

**Published:** 2011-12-23

**Authors:** Guan Ning Lin, Chao Zhang, Dong Xu

**Affiliations:** 1 Department of Computer Science and C.S. Bond Life Sciences Center, University of Missouri, Columbia, MO 65211, USA; 2 Department of Psychiatry, University of California, San Diego, CA 92093, USA

## Abstract

**Background:**

A phylogenetic tree, showing ancestral relations among organisms, is commonly represented as a rooted tree with sets of bifurcating branches (dichotomies) for simplicity, although polytomies (multifurcating branches) may reflect more accurate evolutionary relationships. To represent the true evolutionary relationships, it is important to systematically identify the polytomies from a bifurcating tree and generate a taxonomy-compatible multifurcating tree. For this purpose we propose a novel approach, "PolyPhy", which would classify a set of bifurcating branches of a phylogenetic tree into a set of branches with dichotomies and polytomies by considering genome distances among genomes and tree topological properties.

**Results:**

PolyPhy employs a machine learning technique, BLR (Bayesian logistic regression) classifier, to identify possible bifurcating subtrees as polytomies from the trees resulted from ComPhy. Other than considering genome-scale distances between all pairs of species, PolyPhy also takes into account different properties of tree topology between dichotomy and polytomy, such as long-branch retraction and short-branch contraction, and quantifies these properties into comparable rates among different sub-branches. We extract three tree topological features, 'LR' (Leaf rate), 'IntraR' (Intra-subset branch rate) and 'InterR' (Inter-subset branch rate), all of which are calculated from bifurcating tree branch sets for classification. We have achieved F-measure (balanced measure between precision and recall) of 81% with about 0.9 area under the curve (AUC) of ROC.

**Conclusions:**

PolyPhy is a fast and robust method to identify polytomies from phylogenetic trees based on genome-wide inference of evolutionary relationships among genomes. The software package and test data can be downloaded from http://digbio.missouri.edu/ComPhy/phyloTreeBiNonBi-1.0.zip.

## Background

Evolutionary histories (or phylogenies) are an integral part of many studies in modern biology. Phylogenies have long been used to study the relationships among species. Most phylogenetic trees are in the form of a rooted tree where the leaves represent the species, and internal nodes represent their hypothetical ancestors. Consequently, the phylogenetic tree becomes a branching diagram showing the inferred evolutionary relationships among various biological species based on similarities and differences in their physical and/or genetic characteristics. The two most commonly seen topological sub-structures of trees are dichotomy and polytomy. Although resolving phylogenetic trees into dichotomous branching patterns has been a general goal in phylogenetics [1], the enforced resulting trees might be stretched beyond the necessary evolutionary assumptions.

**Figure 1:**
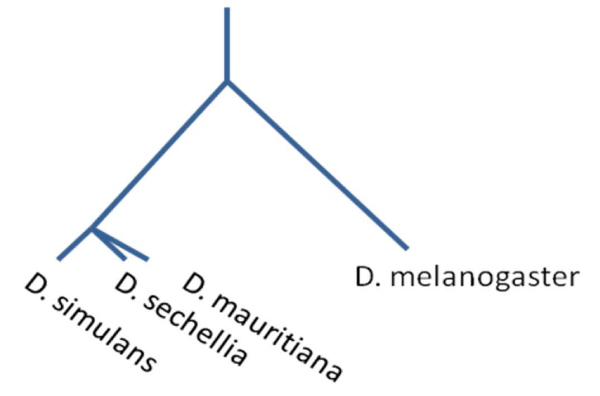
**Phylogenetic tree of *Drosophila melanogaster *species group**. Redrawn from [5]. This phylogeny shows the relationships among some species in the ***Drosophila ***group in which there is a hard polytomy branch. The reason could be that the island species ***D. mauritiana ***and ***D. sechellia ***branched off from the mainland species ***D. simulans ***in a narrow timeframe, such that it is impossible to distinguish which species branched off first and which second.

Polytomies are multifurcating (as opposed to bifurcating) relationships in phylogenetic hypotheses and occur for two reasons: First, polytomies can result from poor resolution of true bifurcating relationships (due to lack of sufficient data or inappropriate analysis of characters), and these are "soft" polytomies; second, polytomies can represent *bona fide *multifurcations (multiple, simultaneous divergence events), and these are "hard" polytomies [2]. For example, simultaneous divergences of three or more lineages can occur due to the isolation of subpopulations within a widespread species by sudden meteorological or geological events resulting in reproductive isolation due to the rapid expansion of the population into open territory. Traditionally, researchers assumed that hard polytomies were exceptions and rare in nature and treated them as expression of ignorance [3,4]. With this assumption, researchers traditionally sought to resolve polytomies in binary trees, often by increasing the number of characters analyzed. However, more recent evidence shows the existence of simultaneous speciation events such as with the *Drosophila simulans *species complex [5,6] shown in Figure 1 and African cichlid fishes which speciate quickly after their home lakes formed in Africa resulting in several phylogenetic polytomies [7].

Microbial taxonomy imposes many difficulties in determining whether generated phylogenetic tree branches are bifurcations or multifurcations. The major problem is that microbes, although morphologically diverse, may have fewer morphological characters than macrobes. Consequently, the rate of change in morphological characters may be greater in macrobes than in microbes [8]. Thus, microbes might not have enough diverse morphologies to do deep taxonomy accurately beyond the level of phylum. As a result, grouping of microbes into many taxonomy trees shows up as multifurcating clads instead of resolved bifurcating trees. Also, bacteria evolve in a different manner from eukaryotes. Eukaryotes predominately evolve by point mutations (changes in single base-pair in DNA) whereas bacteria often evolve by inserting or deleting large chunks of DNA [9-11]. Thus, microbial phylogeny analysis of available molecular sequences, rRNA and protein, have difficulties to convincingly resolve in the many specific branching orders of the microbial divisions due to incongruence of sequence diversity. This suggests that the base of the microbial tree is best seen as a polytomy, an expansive radiation that has not been resolved with current data [12]. As a result, many sources of species trees, such as the NCBI Taxonomy Database, use polytomies for species trees. In fact, 64% of the branch points in the NCBI taxonomy Database [13] have three or more children. In addition, a multifurcated species tree is indeed valuable to infer gene duplication events and orthologs [14-17]. As with any approach that imposes structure on the data [18-20], bifurcations are the imposition of method, not necessarily the reality. Due to the nature of algorithmic pair-wise comparison of many phylogeny methods, often phylogeny visualization tools display a tree as a bifurcating cladogram, which is a directed bifurcating tree with a unique node corresponding to the (usually imputed) most recent common ancestor of all the entities at the leaves of the tree. Thus, all tree-building methods will force a binary tree on the data without considering the possibility that resulting trees might stretch beyond the assumption. A justification may be that it is easier to work on a strictly binary set of nodes although there are possibilities of the polytomies existing in trees [21]. However, even if there is a real dichotomous structure in the data, unresolved nodes will often occur mostly at or near the terminal branches due to lack of information. In this case, it is better to use polytomies instead of forcing dichotomies artificially. As suggested, polytomies might be quite common in microbial taxonomy trees since many times evolutionary relationships of interested species cannot be fully resolved to separate descending branches or difference of timeframes between two divergences.

The most popular approach to obtaining polytomies is through forcing a threshold on bootstrap values of branches generated from a consensus tree, which is produced from a set of gene trees based on different selections of gene subsets. Then multiple bifurcating branches with low bootstrap values would be combined into multifurcating branches. This method has been employed in a number of popular phylogenetic tools, such as PAUP [22], Phylip [23] and Splitstree [22-24]. The result is often *ad hoc *and coarse grain without any significant statistical basis. Another approach for polytomy identification from bifurcating branches is through literature mining and manual curation of biology experts in order to match up with the general understanding of the taxonomy tree of life [5-7] which is laborious and time consuming. Until now, there is still no systematic algorithm and method to automate the process of polytomy identification from a bifurcating tree. Therefore, it is essential to construct a systematic algorithm to identify polytomies from an existing bifurcating tree to facilitate the phylogenetic analysis.

With fast accumulations of molecular sequencing data, especially using whole-genome sequences from different organisms, inferring molecular evolutionary relationships using genomic data has been a popular phylogeny analysis approach in recent years. Often subsets of gene or protein families would be selected to infer the so-called "gene trees" for interested organisms to establish phylogenetic relationships. These analyses typically result in bifurcating phylogeny trees containing only dichotomies. Though gene trees are good sources for hypothesizing species trees, they introduce other problems, such as the incongruence of tree branches in the phylogenetic trees due to different selections of gene subsets. Hence, it is logical to introduce the polytomies instead of trying to resolve every branch when there is incongruence among tree branches of different trees from the same set of species.

In this study, we propose an innovative strategy, called 'PolyPhy' (phylogeny construction with polytomy identification) to identify polytomies from a phylogenetic tree. In PolyPhy, the process starts with phylogenetic tree construction for many species and then combines a feature extraction process with a Bayesian classification method to identify polytomies from a bifurcating phylogenetic tree. The PolyPhy method takes into account different tree topological properties between dichotomy and polytomy, such as long-branch retraction and short-branch contraction, and quantifies these properties as comparable rates among different sub-branches. More specifically, PolyPhy utilizes ComPhy [25], a microbial tree reconstruction tool that we developed based on genome-structure features. After a phylogenetic tree is built, two sets of bifurcating branches, presumably dichotomies and polytomies, are extracted based on Bergeys' Taxonomy. Then three tree structure features, 'LR' (Leaf rate), 'IntraR' (Intra-subset branch rate) and 'InterR' (Inter-subset branch rate), are calculated from bifurcating tree branch sets based on distance matrices derived from ComPhy. The next step is BLR (Bayesian Logistic regression) classification, which is a recursive training process to select best classification parameters to fit the input features and generate a model for classification. The last step is identifying potential polytomies and checking for accuracies.

## Results & discussion

### Optimizing Jackknife ability

**Figure 2:**
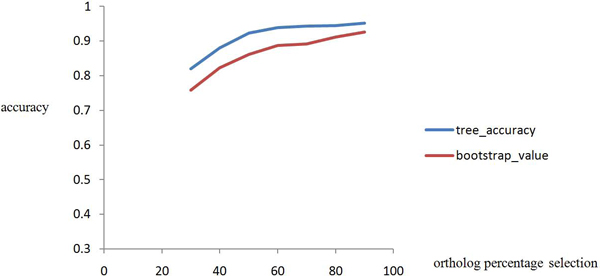
**Accuracy vs. different ortholog percentage selection curves**. The horizontal-axis is percentage (ranging from 30% to 90%) of orthologs being selected to produce the tree and y-axis is the accuracy. This figure shows two different accuracy curves of different ortholog percentage selections. The blue curve shows the accuracy of the tree generated by ComPhy in comparison to the taxonomy. And the red curve is the average bootstrap values from the tree branches for each generated tree.

In ComPhy, genome structures such as breakpoints of gene ordering are constructed to infer genome distance based on orthologs defined in a pair of genomes. Therefore, we treated individual orthologs as samples for jackknife procedure. We resampled the dataset by selecting 30% to 90% of the orthologs incrementally. Figure 2 shows the distribution of average accuracy for trees generated by different percentage selections of orthologs after 50 iterations. It is noticeable that the 60% cutoff worked well since the increase of accuracy beyond this point almost reached a plateau even when more data was included. Therefore, a 60% cutoff was used as the default for genome feature jackknife phylogeny analysis. With 60% jackknife resampling, we could generate unbiased tree replicates for the next step of classification training and testing.

### Classification signal strength in three features

**Figure 3:**
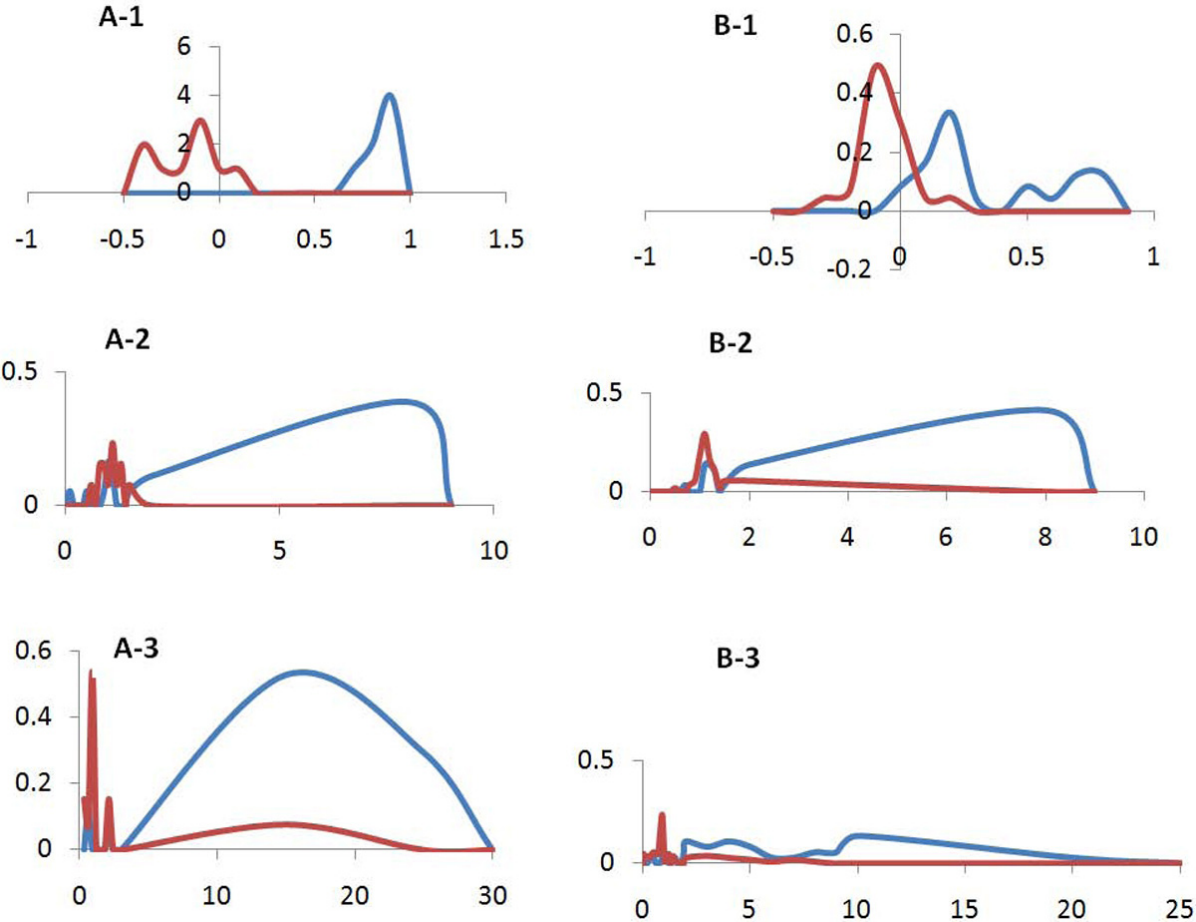
**Classification strength of each of three tree topological features**. Part A of the figures is generated using 83 microbial species based on MSA (multiple sequence alignment) on 16S rRNA sequences. A-1 shows leaf rate topological feature generated per dichotomies and polytomies, while A-2 and A-3 use IntraR and InterR features, respectively. Part B of the figures is generated using all 398 microbial species based on composite distance calculation from ConPhy. B-1 shows leaf rate topological feature generated per dichotomies and polytomies, while B-2 and B-3 use IntraR and InterR features, respectively.

To efficiently utilize each of the features for the classifier described in "Material & Methods," it is important to know how strongly each feature can individually distinguish between dichotomy and polytomy. Figure 3 represents the distribution of each feature value in each class (dichotomy or polytomy) in the two datasets introduced in "Material & Methods." Three features of Part A in Figure 3 are generated from the smaller dataset consisting of 83 genomes; and genome distances were calculated based on traditional MSA (multiple sequence alignment) using 16s rRNA sequences. Part B of Figure 3 is generated from the larger dataset, which has 398 microbial species, and genomes distances were calculated based on composite distance from ComPhy. Figures 3A-1 and 3B-1 correspond to the leaf feature rate distribution, while 3A-2 and 3B-2 correspond to intra-subset branch rate with 3A-3 and 3B-3 from inter-subset branch rate. These figures clearly demonstrate the distinguishable divisions of feature rate distributions between dichotomy and polytomy. Furthermore, similar distribution patterns were observed for all three features between set A figures and set B figures even though these distributions were generated based on different genome distance calculations. This indicates that not only extracted features have strong discerning power, but also the performance is not significantly subject to different genome distance methods.

### BLR parameter optimization

As in other multivariate statistical models, the performance of BLR in classification depends on the combination of several parameters. Here, BLR involves mostly two classes of parameters: 1) the prior variance parameter *V *and 2) the error tuning parameter *t. V *is a regulation parameter that controls the balance and tradeoff among individual feature distribution variance and their prior skews; *V *also tries to minimize the overall distribution variance. How to choose a good set of prior variance is not well understood and varies from dataset to dataset. Therefore, we chose variance (*V*) values based on commonly used variance range and applied the best fit. For *V*: *V_i+1 _*= 2*V_i _, where *V_1 _*= 0.002 and *i *= 1,..., 20, *i *is the number of iterations. The error tuning *t *is another parameter to help confirm the right selection of other parameters by trying different accuracy measurements. Five criteria can be used for error tuning of *t *with 0 representing a no tuning threshold:

1) sum of error = (FP + FN);

2) balanced error rate BER = (sensitivity + specificity)/2;

3) T11U = 2*TP - FP [26];

4) F1 measure [27];

5) T13U = 20*TP - FP [28], where *TP *is true positive value, *FP *is false positive value and *FN *is false negative value.

Accordingly, two parameters, *V *and *t*, should be optimized. The parameter optimization was performed by using a grid search within a limited range. To minimize over-fitting of the prediction model, three-fold crossover validation was used to investigate the training set. To evaluate predicted accuracy, an *F*-measure was used [27] as the weighted harmonic mean of precision and recall; and it is defined as follows:

$$F1(F-\text{measure)=2*}\frac{precision*recall}{precision+recall}=\frac{2*TP}{2*TP+FP+FN}.$$

**Figure 4:**
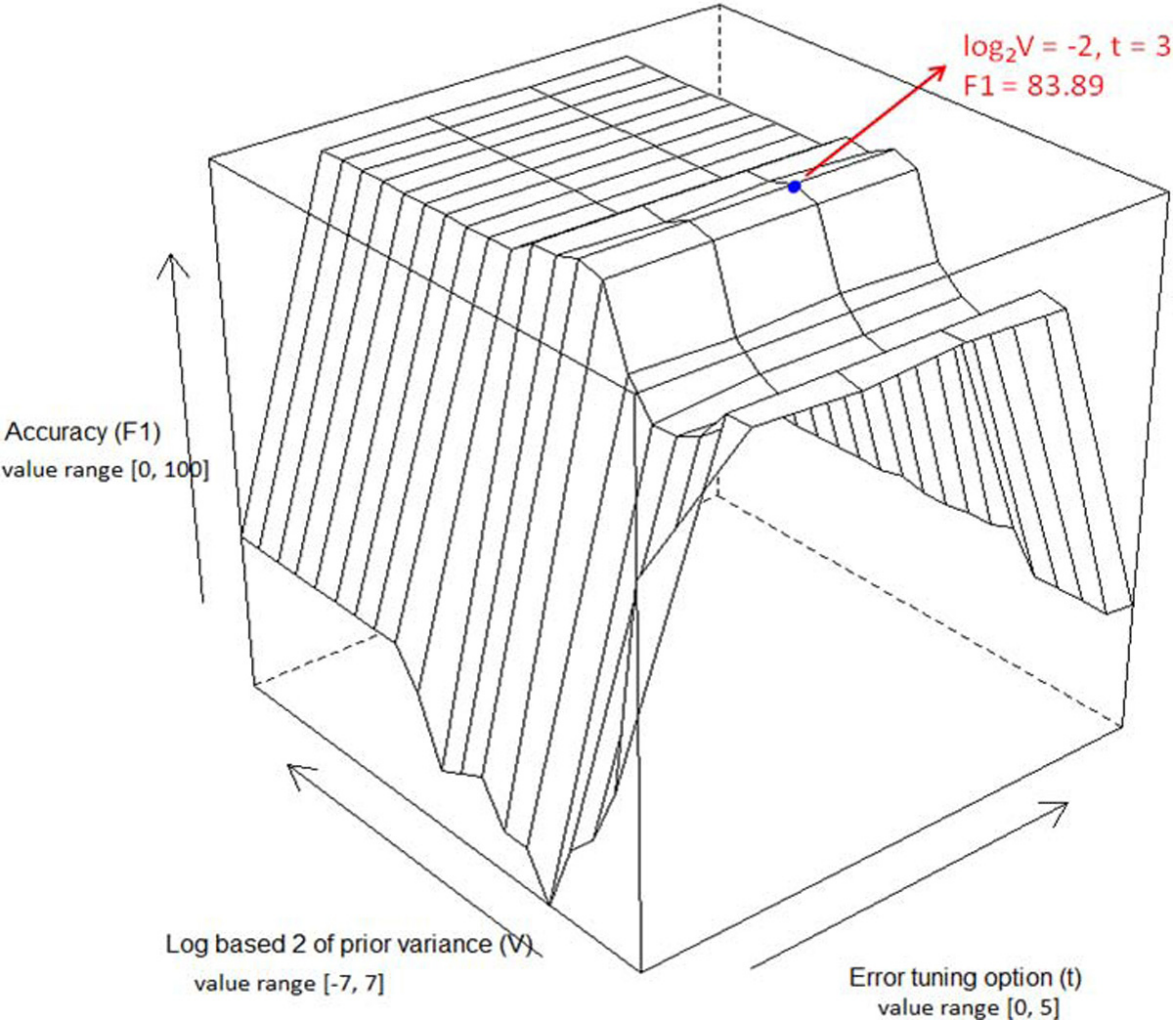
**Accuracy surface**. Accuracy surface of threefold crossover validation on training set versus the variations of parameters ***log_2_***(***V) ***(logged prior variance) and ***t ***(error tuning option). The blue point represents the highest accuracy peak on the surface.

During the BLR classification model training, each data point represents a 2-level bifurcation with three leaf nodes (a triplet). If the bifurcation triplet is in the gold standard dichotomy set from Bergey tree, one must assign class label 1; otherwise -1. Figure 4 shows the profile of predicting the accuracy of the three-fold cross-validation on the training set versus the variations of the parameters *V *and *t*. Obviously, the accuracy profile has a maximum values peak at (*V*, *t*) = (0.128, 3), indicating that the optimal values of V and t for constructing BLR models are 0.128 and 3, respectively.

Another parameter we provide in the PolyPhy algorithm is to let users choose types of priors for BLR classification. Two types of prior are commonly employed, Laplace (sparse data and less features) and Gaussian (approximate normal distribution). Users can try both prior types to achieve the best prediction result based on their data property.

### Prediction ability

Using the optimal values of *V *and *t*, the polytomy classification model was constructed based on the training set by using the BLR learning algorithm provided by the BBR package [29]. To minimize data dependence on the prediction model, 10 tree replicates were prepared through jackknife procedure, five from each dataset (see "Material & Methods"). Each training set from data set 1 consisted of around 200 Bergey system confirmed bifurcating triplets, while each training set from data set 2 consisted of around 60 confirmed bifurcating triplets. All training sets were half confirmed dichotomies and half confirmed polytomies. The classification results are listed in Table 1. For all test models, the balanced accuracies were > 80.24%, and the AUC value was 83.31%. Training sets from data set 2 had higher accuracy than sets from data set 1 because of their less variance from the smaller genome set.

**Table 1:** Classification results of the test sets

	Test set	Accuracy, %	Precision, %	Recall, %	ROC AUC value
	data1.1	81.34	75.55	88.1	87.89
	data1.2	83.38	79.15	88.09	89.39
	data1.3	80.24	88.39	73.47	85.71
	data1.4	80.29	89.89	72.55	88.09
	data1.5	80.49	70.67	93.48	83.31
	
combined	data1	81.15	80.73	83.138	86.88

	data2.1	88.24	88.24	88.24	92.52
	data2.2	86.09	85.36	86.84	91.01
	data2.3	89.21	90.01	88.42	94.52
	data2.4	87.98	87.24	88.74	92.54
	data2.5	83.23	84.24	82.24	85.12
	
combined	data2	86.95	87.02	86.90	90.74

Precision is the true positive/(true positive + false positive). Recall is true positive/(true positive + false negative). AUC quantifies the overall ability of the test to discriminate between positive samples and negative samples.

To further improve the classification accuracy, three topological features were added per different distance to the BLR classifier. Distance measures GDD, GBD and GCD from ComPhy were used with all three tree features included per distance respectively (nine features in total). The classification accuracy reached as high as 0.87 for balanced accuracy, 0.95 precision, 0.85 recall, and 0.95 AUC value for all the training sets from data set 1, but the classification accuracy showed only around 5% improvement for training sets from data set 2.

### Multifucation tree generation

**Figure 5:**
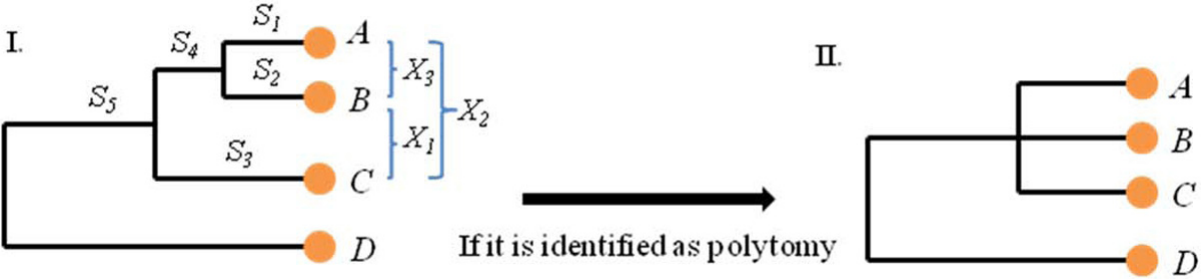
**Polytomy identification**. Part I presents a three-level bifurcation subtree with 4 genome taxa ***A***, ***B***, ***C ***and ***D***. ***X_1_***, ***X_2 _***and ***X_3_***are genome distances calculated among ***A***, ***B ***and ***C***. And ***S_1_***, ***S_2_***, ***S_3_***, ***S_4_***and ***S_5_***are branch lengths. If bifurcating subtree of ***A***, ***B ***and ***C ***is identified as a polytomy, then the sub-tree can be converted to a multifurcation subtree, shown as Part II.

With polytomies being identified through the BLR classification, PolyPhy can make a multifurcating tree. The PolyPhy team took the most intuitive approach by using the average of the branch lengths from a bifurcation identified as a polytomy. For example, from Figure 5, Part I, where *S_1_*, *S_2_*, *S_3 _*and *S_4 _*are branch lengths from a bifurcation, *S_1_*, *S_2 _*and *S_3 _*were averaged and the branch *S_4 _*was removed in order to make the subtree like Part II in Figure 5. Note, in the classification process, PolyPhy generated either independent classifier for each jackknife-generated tree or a combined classifier for one overall tree to accommodate those who just want to see one final tree produced in the end when doing analysis. Therefore, in generating the final tree, a consensus multifurcating tree was produced from several independent multifurcating trees and multiple classifiers were applied. We applied the accuracy evaluation from ComPhy [25] and evaluated the accuracies of the multifurcating trees against Bergey's taxonomy system. A comparable accuracy was achieved with 30% more polytomies being validated due to the fact that ComPhy previously only evaluated dichotomy branches.

## Conclusion

Polyphy, a standalone tool for phylogeny reconstruction and polytomy identification, is robust and easy to use for studying evolution. It can construct a consensus tree without requiring multiple sequence alignment and can also identify polytomies without requiring manual interferences. It is a fully automated tool. The phylogeny reconstruction implements a jackknife recursive sampling procedure; then recursively trains the BLR classification model for polytomy identification. It allows users to infer a multifurcating phylogenetic tree for any set of microbial genomes of interest to study their evolutionary relationships. Although the tool is built for reconstructing a multifurcating phylogeny from whole genome sequences, users can have a pre-generated bifurcating tree from any phylogenetic tool as the starting point for classification. We believe that this is a timely and necessary development as more and more microbes are sequenced daily (especially from the metagenomic analysis of microbial communities) without reliable taxonomy established.

## Material & methods

### Taxon selections

We used a set of 398 single-chromosome microbial genomes, as shown in Table 2, consisting of 31 Archaea and 367 bacterial genome sequences. These sequences were previously used in the development of ComPhy [25]. The distribution of phylum clad in this dataset represents the trend of microbial taxonomy classification [30], in which most genome sequences are from three phyla, *Proteobacteria*, *Firmicutes *and *Actinobacteria*.

**Table 2:** Taxon statistics of the 432 prokaryotic complete genomes

Phylum	C	O	F	G	S	str
A1	1	3	4	4	7	7
A2	8	9	12	18	23	23
A3	1	1	1	1	1	1

Subtotal 3	10	13	17	23	31	31

B1	1	1	1	1	1	1
B2	1	1	1	1	1	1
B4	1	2	2	2	3	4
B6	1	1	1	1	2	2
B10	1	3	3	8	15	19
B11	1	1	1	2	4	4
B12	5	33	53	99	157	208
B13	3	7	14	22	58	96
B14	3	9	15	16	31	35
B15	1	1	1	1	1	1
B16	1	1	2	3	7	11
B17	1	1	2	3	7	9
B19	1	1	1	2	2	2
B20	3	3	5	5	6	7
B21	1	1	1	1	1	1

Subtotal 15	25	66	103	167	296	401

Total 18	35	79	120	190	327	432

P = Phylum, C = Class, O = Order, F = Family, G = Genus, S = Species, str = strain. A = Archaea and B = Bacteria.

The second dataset consists of 83 microbial genomes [31], whose 16S rRNA sequences were obtained from the Greengenes microbial database [32]. These 83 genomes are also part of the first data set of 398 microbial genomes, and they were used to generate phylogenetic tree solely based on the 16S rRNA sequences.

### PolyPhy (phylogeny with polytomy identification) tool

This research developed a machine-learning tool, PolyPhy, for polytomy identification. PolyPhy starts with ComPhy reconstructing a large-scale microbial phylogeny using whole-genome structural features. It obtains multiple unbiased trees through a jackknife procedure. PolyPhy uses three tree topological features to be discussed in following sections, and through recursive trainings using a machine learning method to identify polytomy from the tree. The last step of the tool is generating a meaningful multifurcating tree. No fixed threshold is imposed on any of the patterns to decide if a two-level bifurcation is a dichotomy or polytomy since every phylogenetic tree will have a different distance value and branch. Thus, through a machine-learning method, i.e., the BLR classifier, the extracted features can be clearly used to train the classification model without needing to know the exact cutoff values.

### Phylogenetic tree reconstruction

Obtaining an optimal binary phylogenetic tree as input tree for PolyPhy is as important as identifying polytomy from the binary tree. It has been traditionally considered that likelihood-based methods have more accurate phylogenetic relationship inferences than distance-based methods such as Neighbor-Joining (NJ) [33,34]. However, in 2010, Roch [35] showed that when sets of large-scale species are involved in phylogenetic analysis, the distance-based method proved much more effective, practical and surprisingly comparable in performance [36]. Thus, given the amount of genomes used in this study, distance-based phylogenetic inference was applied.

### Phylogenetic tree reconstruction using ComPhy

ComPhy [25] is a stand-alone Java-based tool which utilizes a robust and efficient strategy called 'Gene Composite Distance' to combine different aspects of evolutionary relationships among genomes for producing a phylogenetic tree from a given set of whole genome sequences. Specifically, composite distance measure starts with an all-against-all pairwise genome comparison using BLASTP [37]. In the second step, a distance matrix is calculated from three components, i.e., GDD (Gene Dispersion distance), GBD (Genome Breakpoint distance) and GCD (Gene Content distance). This distance matrix is then fed into a distance-based algorithm, Neighbor-Joining (NJ) [33,34], using a third-party tool, Phylip [23], to produce phylogenetic trees.

### Phylogenetic tree reconstruction using 16s rRNA sequences

Besides using the whole genome structural features to infer microbial phylogeny by ComPhy, we also applied multiple-sequence alignment using 16s rRNA sequences from the second dataset of microbial genome (see more detail in Taxa selections section) for evolution distance inference. MUSCLE [38] was applied for multiple sequence alignments for datasets with the 83 microbes. Then a pairwise genome distance matrix was generated from the alignments using "distMat" from EMBOSS with Kimura correction [39]. Finally, this distance matrix was fed into Neighbor-Joining (NJ) [33,34], using Phylip [23] to produce a phylogenetic tree.

### Jackknife procedure in phylogeny

Jackknifing is a statistical method to estimate the accuracy of sample statistics by repeatedly using subsets of available data to produce the results. In order to obtain multiple meaningful phylogenetic microbial trees, PolyPhy provides the user with capability to generate trees from the same dataset through a jackknife procedure to obtain robust and non-biased training datasets of bifurcating subtrees from the trees. We subsequentially selected different subsets of whole genome gene sets in which ortholog ordering was the main component used in ComPhy distance measure to generate different trees. It is not a trivial task to apply the perturbation to ortholog selection and assess the robustness of the data. Though some have tried to use jackknife in large-scale phylogeny analysis, the studies were not conclusive [40,41]. Here, we conducted some of the performance tests on jackknife procedure using 398 microbial genomes.

The major steps are performed as follows: 1) Generating a new set of orthologs by randomly selecting *k% *from overall orthologs of each genome. Orders of the selected orthologs are preserved with respect to their order in the original genomes. 2) Reconstructing a tree replicate from these genomes with new ortholog subsets. 3) Repeat step 1 with same *k *value but different randomly chosen ortholog subsets 50 times to obtain 50 different tree replicates. 4) Compute a consensus tree and corresponding confidence values on all internal branches by using the CONSENSE program in PHYLIP [23] from 50 tree replicates. 5) Repeat Step 1 with different *k *values, ranging from 30 to 80. Through this procedure, an optimal selection of subset of orthologs can be determined for jackknife selection.

### Polytomy identification through classification

We have developed a machine-learning process to carry out the task of identifying polytomies from given bifurcation tree. Figure 5.I is an example of the bifurcating tree with three levels of bifurcating branching, (((*A*, *B*), *C*), *D*). If evolutionary relationships among leaves genomes *A*, *B *and *C *cannot be fully resolved due to conflict supports with low bootstrap values or simply more likely simultaneous speciation, then we would classify this bifurcating branch as a polytomy subtree, as shown in Figure 5.II. We will utilize the BLR (Bayesian Logistic Regression) classification, a machine learning approach, to automatically learn and classify bifurcating branches into dichotomies or polytomies. After a classification model is trained from extracted features, we can classify a bifurcating branch into a dichotomy subtree or a polytomy subtree. The following shows the details of the procedure:

#### (1) Bayesian logistic regression

The Bayesian approach is attractive for its ability to incorporate prior knowledge into statistical inference; and the logistic regression can provide a simple but accurate model for the predictions with calibrated probabilities without extrapolating input datasets to a complex and higher dimension. Bayesian logistic regression utilizes the best features of both methods by employing the prior information about the success probability and recursively optimizing the prediction parameters to achieve the optimal classification/prediction rather than simple regression coefficient for classifiers [42-44]. BBR (Bayesian Logistic Regression Software) package [29] was used to train the model and classify the bifurcations for dichotomies and polytomies.

#### (2) Classification training datasets

The format of training data for classification is comprised of various two-level bifurcating subtrees as shown in Figure 5.I. The gold standard for deciding whether or not a binary tree branching is a dichotomy or a polytomy is the tree topology provided by Bergey's microbial taxonomy classification system. For 398 microbial taxa, we constructed a bifurcating phylogenetic tree and were able to extract 370 dichotomies and 285 polytomies as training data. The dataset was split into 90% for model training and 10% for testing the accuracy of the prediction model.

#### (3) Classification features

**Figure 6:**
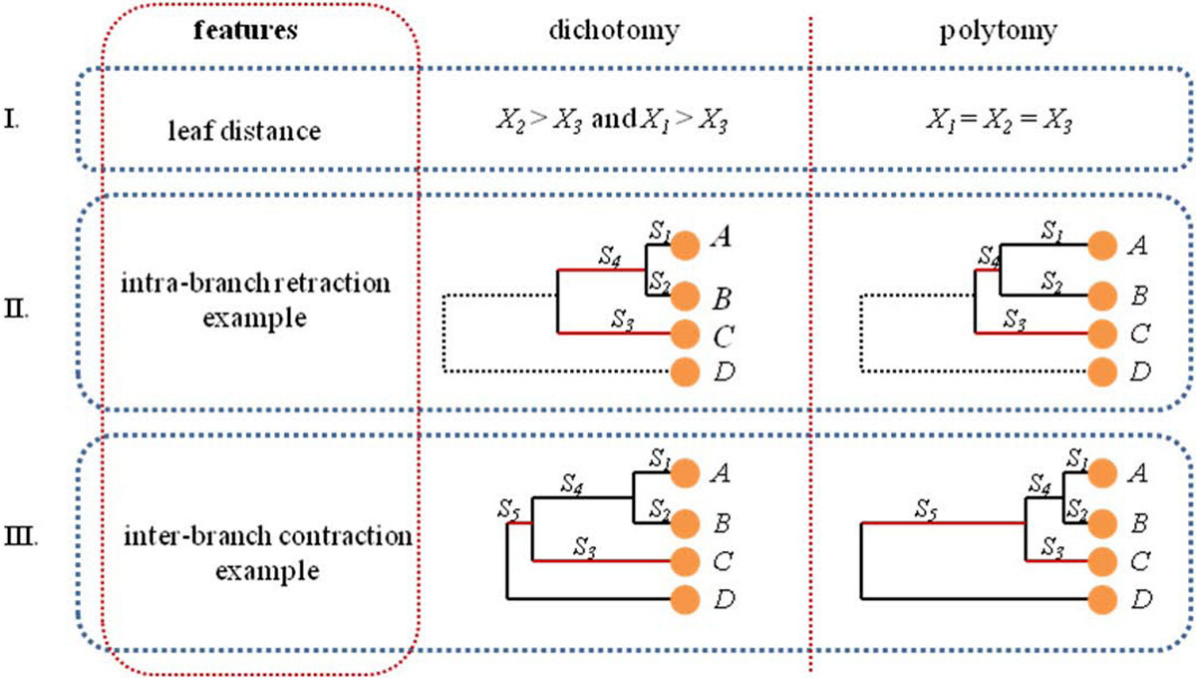
**Polytomy identification using three different features**. This graph presents three different topological tree structure features for the BLR classification. Part I, II and III show examples on how to calculate different values for each of three features for dichotomy bifurcation and polytomy bifurcation; and also how different branch contractions and retractions are presented in subtrees.

In order to successfully train a classification model for bifurcating branches from a phylogenetic tree, there must be a meaningful set of features extracted from the bifurcating branches in a tree. Figure 5.I gives a hypothetical bifurcation (a two-layer binary subtree in phylogram format) with four different genomes, *A*, *B*, *C *and *D *as leaf nodes, in which genome *D *is used as out-group taxa. Genome distances among *A*, *B *and *C *are represented as *X_1_*, *X_2 _*and *X_3_*, which can be calculated from ComPhy or any other phylogeny construction tool preferred by the users. The branch lengths, *S_1_*, *S_2_*, *S_3_*, *S_4 _*and *S_5 _*are usually the branch lengths generated by different tree generation algorithms. With this information given by tree topology, three topological features can be generated which are consistent and can be extracted from any bifurcating branch of the tree. They are LR (Leaf rate) as Figure 6.I, IntraR (Intra-subset branch rate) as Figure 6.II and InterR (inter-subset branch rate) as Figure 6.III.

The common misconception of the branch length in phylogenetic tree is that it is an irrelevant factor for inferring species branching history compared to other types of topology information such as hierarchy of the branches. Although this view could simplify the presentation of the tree and still give the users a somewhat coarse and high-level examination to infer the evolution history using ordering of the taxa, the sensitivity of relative evolution timing is lost due to this assumption. For the next two topological features, branch length must be considered as a strong supporting factor for evolution history inferences. The correct branch length is not a random assignment, but rather a statistical derivation from different phylogenetic tools proportional to the predicted or hypothetical evolutionary time between organisms or sequences based on the presumed evolution model [45-47]. Therefore, the two patterns, IntraR and InterR extracted from the tree, use the idea of long-branch retraction and short-branch contraction for polytomy identification.

#### (4) Feature LR generation

This pattern studies the relative genome distances among three studied genomes. In Figure 6.I, if the bifurcations is a dichotomy, then *X_3_*, distance between genome *A *and genome *B*, will be the shortest genome distance among all three (*X_1_*, *X_2 _*and *X_3_*). Therefore, the summation of distance of *A *to *C *and *B *to C will always be greater than twice the distance of *A *to *B*. Also by normalizing with the average distance, this pattern can indicate the difference between a dichotomy and a polytomy: $\text{Leaf rate}=\frac{X_{1}+X_{2}-2*X_{3}}{\left(X_{1}+X_{2}+X_{3}\right)∕3}$, where *X_1_*, *X_2 _*and *X_3 _*are pair-wise genome distances among *A*, *B *and *C *(see Figure 6.I).

#### (5) Feature IntraR generation

This feature considers all the branches within the two-level bifurcation (Figure 6.II). If one assumes that the two-levels of branching from the top parent node in a given bifurcation correspond to two different steps of speciation, which is dichotomy, then one should see a clear separation between speciation of *C *and set of (*A*, *B*), and between *A *and *B*. Therefore, the branch of *S_4 _*attempts to retract and stretch as far as possible to have the same branch length of *S_3_*. On the contrary, if this subtree of bifurcation is indeed a polytomy, then length of the branch *S_4 _*will be as short as possible so that the length *S_1 _*will be similar to that of *S_2 _*in order to have simultaneous speciation. Hence, the following formula captures the contraction and retraction properties and uses them to identify dichotomy and polytomy: $\text{Intra}-\text{subset branch length}=\frac{\text{min}\left(S_{1},S_{2}\right)∕\text{max}\left(S_{1},S_{2}\right)}{\text{min}\left(S_{3},S_{4}\right)∕\text{max}\left(S_{3},S_{4}\right)}$, where *S_1_*, *S_2_*, *S_3 _*and *S_4 _*are the branch lengths. *S_1 _*and *S_2 _*could have the same length, which has no impact on the formula.

#### (6) InterR generation

This last classifier feature covers the branch relationship between the two-level bifurcation and its level above through branch s5, as shown in Figure 6.III. If one assumes species *A*, *B *and *C *speciated at the same time, then the branch contraction property will force all branches including *S_1_*, *S_2 _*and *S_3 _*to have similar lengths of branching time for species *A*, *B *and *C *to be clustered together into a polytomy. With another branch *S_5 _*from one higher level of hierarchic topology, it is easier to see that the contraction of *S_4 _*against *S_5 _*has an observable longer branch length. Most dichotomies show that *S_5 _*is shorter than at least one of the intra-subset branches, while *S_5 _*is much longer than all of the intro-subset branches for a polytomy. Thus: $\text{inter}-\text{subset branch length}=\frac{\text{max}\left(S_{1},S_{2},S_{3},S_{4}\right)}{S_{5}}$, where *S_1_*, *S_2_*, *S_3_*, *S_4 _*and *S_5 _*are the branch lengths.

#### (7) Classification procedure

For BLR classifier, properly estimating the posteriori probability is the crucial step in the process. In order to have the maximum *posteriori *estimate of the selected class label for input data, an optimal value of prior variance is needed through a rigorous training. In PolyPhy, the recursive prior variance selection can be performed within a range of values through *k*-fold cross-validation to obtain the optimal parameter settings for the training model. For each prior variance value, BLR trains *k *logistic regression models under *a prior *with that hyperparameter. Each model is trained on the union of *k-1 *of the subsets and tested on the remaining subset with each subset used as the test subset once. BLR then selects the prior variance that maximizes the average value of the log-likelihood of a training instance when it appears in the test subset.

There are two ways to use the BLR classifier. One is the individual tree training and prediction. From each jackknife generated bifurcating tree, PolyPhy extracts all bifurcating subtrees. The gold standard matched dichotomies and polytomies are used as input to the classifier on the rest subsets. The best classification model is trained through 3-fold cross validation and obtained for each tree. The classifier is then applied to the rest of the bifurcating subtrees for each tree to identify the polytomies and convert those bifurcating branches into polytomies. Subsequently a consensus tree can be obtained from all the multifurcating trees. With this final tree, the result tree can be compared to bifurcating tree or consensus tree obtained from multiple bifurcating trees. Another simpler way of using the BLR classifier is to combine all the generated trees to create an overall consensus tree and extract one set of bifurcating subtree input based on the gold standard Bergey bifurcation set. Next a classifier model is generated to classify the rest of the bifurcating subtrees for each tree to identify the polytomies and convert those bifurcating branches into polytomies.

## Availability

**PolyPhy and all the relevant resources are available at **http://digbio.missouri.edu/ComPhy/phyloTreeBiNonBi-1.0.zip

## Competing interests

The authors declare that they have no competing interests.

## Authors' contributions

GNL carried out the algorithm constructions, testing and drafted the manuscript. ZC organized the java codes and made them available to public. DX conceived and coordinated the study. All authors have read, modified and approved the final manuscript.

## References

[B1] Rokas A Carroll SB Bushes in the tree of life PLoS Biol 2006 4 e352 10.1371/journal.pbio.0040352 17105342 PMC1637082

[B2] Maddison DR Reconstructing character evolution on polytomous cladograms Cladistics 1989 5 365 377 10.1111/j.1096-0031.1989.tb00569.x 34933477

[B3] Felsenstein J Phylogenies and the comparative method Am Nat 1985 1 15 10.1086/284325 31094602

[B4] Grafen A The phylogenetic regression Philos Trans R Soc Lond B Biol Sci 1989 326 119 157 10.1098/rstb.1989.0106 2575770

[B5] Coyne JA Elwyn S Kim SY Llopart A Genetic studies of two sister species in the Drosophila melanogaster subgroup, D. yakuba and D. santomea Genet Res 2004 84 11 26 10.1017/S0016672304007013 15663255

[B6] Kliman RM Andolfatto P Coyne JA Depaulis F Kreitman M Berry AJ McCarter J Wakeley J Hey J The population genetics of the origin and divergence of the Drosophila simulans complex species Genetics 2000 156 1913 1931 11102384 10.1093/genetics/156.4.1913 PMC1461354

[B7] Takahashi K Terai Y Nishida M Okada N Phylogenetic relationships and ancient incomplete lineage sorting among cichlid fishes in Lake Tanganyika as revealed by analysis of the insertion of retroposons Mol Biol Evol 2001 18 2057 2066 11606702 10.1093/oxfordjournals.molbev.a003747

[B8] Taylor JW Turner E Townsend JP Dettman JR Jacobson D Eukaryotic microbes, species recognition and the geographic limits of species: examples from the kingdom Fungi Philos Trans R Soc Lond B Biol Sci 2006 361 1947 1963 10.1098/rstb.2006.1923 17062413 PMC1764934

[B9] Hedlund BP Staley JT Phylogeny of the genus Simonsiella and other members of the Neisseriaceae Int J Syst Evol Microbiol 2002 52 1377 1382 10.1099/ijs.0.01952-0 12148653

[B10] Hugenholtz P Goebel BM Pace NR Impact of culture-independent studies on the emerging phylogenetic view of bacterial diversity J Bacteriol 1998 180 4765 4774 9733676 10.1128/jb.180.18.4765-4774.1998 PMC107498

[B11] Hillis DM Taxonomic sampling, phylogenetic accuracy, and investigator bias Syst Biol 1998 47 3 8 10.1080/106351598260987 12064238

[B12] Pace NR The large-scale structure of the Tree of Life Microbial Phylogeny and Evolution: Concepts and Controversies 2005 53 69

[B13] Wheeler DL Barrett T Benson DA Bryant SH Canese K Church DM DiCuccio M Edgar R Federhen S Helmberg W Database resources of the National Center for Biotechnology Information Nucleic Acids Res 2005 33 D39 45 10.1093/nar/gki062 15608222 PMC540016

[B14] Chiu JC Lee EK Egan MG Sarkar IN Coruzzi GM DeSalle R OrthologID: automation of genome-scale ortholog identification within a parsimony framework Bioinformatics 2006 22 699 707 10.1093/bioinformatics/btk040 16410324

[B15] Li H Coghlan A Ruan J Coin LJ Heriche JK Osmotherly L Li R Liu T Zhang Z Bolund L TreeFam: a curated database of phylogenetic trees of animal gene families Nucleic Acids Res 2006 34 D572 580 10.1093/nar/gkj118 16381935 PMC1347480

[B16] Ruan J Li H Chen Z Coghlan A Coin LJ Guo Y Heriche JK Hu Y Kristiansen K Li R TreeFam: 2008 Update Nucleic Acids Res 2008 36 D735 740 10.1093/nar/gkm1005 18056084 PMC2238856

[B17] Simmons MP Freudenstein JV Uninode coding vs gene tree parsimony for phylogenetic reconstruction using duplicate genes Mol Phylogenet Evol 2002 23 481 498 10.1016/S1055-7903(02)00033-7 12099800

[B18] Maddison W Reconstructing character evolution on polytomous cladograms Cladistics 1989 5 365 377 10.1111/j.1096-0031.1989.tb00569.x 34933477

[B19] Hoelzer GA Meinick DJ Patterns of speciation and limits to phylogenetic resolution Trends Ecol Evol 1994 9 104 107 10.1016/0169-5347(94)90207-0 21236789

[B20] Whitfield JB Lockhart PJ Deciphering ancient rapid radiations Trends Ecol Evol 2007 22 258 265 10.1016/j.tree.2007.01.012 17300853

[B21] Chan KM Moore BR Symmetree: whole-tree analysis of differential diversification rates Bioinformatics 2005 21 1709 1710 10.1093/bioinformatics/bti175 15572466

[B22] Wilgenbusch JC Swofford D Inferring evolutionary trees with PAUP* Curr Protoc Bioinformatics 2003 Chapter 6 Unit 6 4 10.1002/0471250953.bi0604s00 18428704

[B23] Felsenstein J PHYLIP -- Phylogeny inference package (Version 3.2) Cladistics 5 1989 164 166

[B24] Huson DH Bryant D Application of phylogenetic networks in evolutionary studies Mol Biol Evol 2006 23 254 267 10.1093/molbev/msj030 16221896

[B25] Lin GN Cai Z Lin G Chakraborty S Xu D ComPhy: prokaryotic composite distance phylogenies inferred from whole-genome gene sets BMC Bioinformatics 2009 10 Suppl 1 S5 10.1186/1471-2105-10-S1-S5 19208152 PMC2648732

[B26] Soboroff I Robertson S Building a filtering test collection for TREC 2002 Proceedings of the 26th Annual International ACM SIGIR Conference on Research and Development in Informaion Retrieval 2003

[B27] Rijsbergen v J C Information Retrieval Butterworth 1979 2

[B28] Hersh W Voorhees E TREC genomics special issue overview Information Retrieval 2009 12 1 15 10.1007/s10791-008-9076-6

[B29] de Chassey B Navratil V Tafforeau L Hiet MS Aublin-Gex A Agaugue S Meiffren G Pradezynski F Faria BF Chantier T Hepatitis C virus infection protein network Mol Syst Biol 2008 4 230 10.1038/msb.2008.66 18985028 PMC2600670

[B30] Hugenholtz P Exploring prokaryotic diversity in the genomic era Genome Biol 2002 3 REVIEWS0003 10.1186/gb-2002-3-2-reviews0003 11864374 PMC139013

[B31] Henz SR Huson DH Auch AF Nieselt-Struwe K Schuster SC Whole-genome prokaryotic phylogeny Bioinformatics 2005 21 2329 2335 10.1093/bioinformatics/bth324 15166018

[B32] DeSantis TZ Hugenholtz P Larsen N Rojas M Brodie EL Keller K Huber T Dalevi D Hu P Andersen GL Greengenes, a chimera-checked 16S rRNA gene database and workbench compatible with ARB Appl Environ Microbiol 2006 72 5069 5072 10.1128/AEM.03006-05 16820507 PMC1489311

[B33] Saitou N Nei M The neighbor-joining method: a new method for reconstructing phylogenetic trees Mol Biol Evol 1987 4 406 425 3447015 10.1093/oxfordjournals.molbev.a040454

[B34] Studier JA Keppler KJ A note on the neighbor-joining algorithm of Saitou and Nei Mol Biol Evol 1988 5 729 731 3221794 10.1093/oxfordjournals.molbev.a040527

[B35] Roch S Toward extracting all phylogenetic information from matrices of evolutionary distances Science 2010 327 1376 1379 10.1126/science.1182300 20223986

[B36] Allman ES Rhodes JA Evolution. Trees, fast and accurate Science 2010 327 1334 1335 10.1126/science.1187797 20223973

[B37] Lopez R Silventoinen V Robinson S Kibria A Gish W WU-Blast2 server at the European Bioinformatics Institute Nucleic Acids Res 2003 31 3795 3798 10.1093/nar/gkg573 12824421 PMC168979

[B38] Edgar RC MUSCLE: multiple sequence alignment with high accuracy and high throughput Nucleic Acids Res 2004 32 1792 1797 10.1093/nar/gkh340 15034147 PMC390337

[B39] Rice P Longden I Bleasby A EMBOSS: the European Molecular Biology Open Software Suite Trends Genet 2000 16 276 277 10.1016/S0168-9525(00)02024-2 10827456

[B40] Belda E Moya A Silva FJ Genome rearrangement distances and gene order phylogeny in gamma-Proteobacteria Mol Biol Evol 2005 22 1456 1467 10.1093/molbev/msi134 15772379

[B41] Luo H Shi J Arndt W Tang J Friedman R Gene order phylogeny of the genus Prochlorococcus PLoS One 2008 3 e3837 10.1371/journal.pone.0003837 19050756 PMC2585141

[B42] Strimenopoulou F Brown PJ Empirical Bayes logistic regression Stat Appl Genet Mol Biol 2008 7 Article9 10.2202/1544-6115.1359 18312223

[B43] Clark TG De Iorio M Griffiths RC Bayesian logistic regression using a perfect phylogeny Biostatistics 2007 8 32 52 10.1093/biostatistics/kxj030 16556611

[B44] Cawley GC Talbot NL Gene selection in cancer classification using sparse logistic regression with Bayesian regularization Bioinformatics 2006 22 2348 2355 10.1093/bioinformatics/btl386 16844704

[B45] Yang Z Rannala B Branch-length prior influences Bayesian posterior probability of phylogeny Syst Biol 2005 54 455 470 10.1080/10635150590945313 16012111

[B46] Venditti C Meade A Pagel M Detecting the node-density artifact in phylogeny reconstruction Syst Biol 2006 55 637 643 10.1080/10635150600865567 16969939

[B47] Francois O Mioland C Gaussian approximations for phylogenetic branch length statistics under stochastic models of biodiversity Math Biosci 2007 209 108 123 10.1016/j.mbs.2007.01.005 17350052

